# [4-(4-Meth­oxy­phen­yl)-1-methyl-3-nitro­pyrrolidin-3-yl]methanol

**DOI:** 10.1107/S1600536813003073

**Published:** 2013-02-09

**Authors:** K. Prathebha, S. Sathya, G. Usha, N. Sivakumar, M. Bakthadoss

**Affiliations:** a PG and Research Department of Physics, Queen Mary’s College, Chennai-4, Tamilnadu, India; b Department of Organic Chemistry, University of Madras, Guindy Campus, Chennai-25, Tamilnadu, India

## Abstract

In the title compound, C_13_H_18_N_2_O_4_, the dihedral angle between the benzene and pyrrolidine (all atoms) rings is 70.6 (1)°. The pyrrolidine ring adopts a half-chair conformation. In the crystal, mol­ecules form chains along the *c*-axis direction linked by O—H⋯N hydrogen bonds, which are then connected by C—H⋯O inter­actions, forming a sheet parallel to the *bc* plane.

## Related literature

For information on the pyrrolidine ring in biologically active natural compounds, see: Gu *et al.* (2004 ▶). For the use of pyrrolidine-containing mol­ecules in the treatment of diseases, see, for example: Horri *et al.* (1986 ▶) for diabetes and Karpas *et al.* (1988 ▶) for viral infections. For bond lengths in a related structure, see: Jayabharathi *et al.* (2009 ▶).
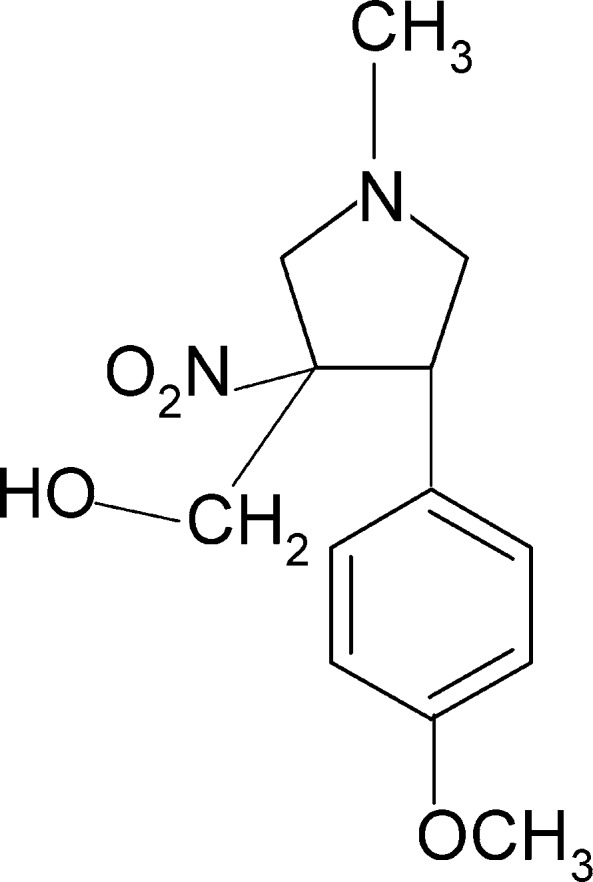


## Experimental

### 

#### Crystal data

C_13_H_18_N_2_O_4_
*M* *_r_* = 266.29
Monoclinic, 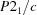

*a* = 11.6827 (10) Å
*b* = 11.1912 (11) Å
*c* = 11.1789 (11) Å
β = 109.118 (2)°
*V* = 1381.0 (2) Å^3^
*Z* = 4
Mo *K*α radiation
μ = 0.10 mm^−1^
*T* = 293 K
0.22 × 0.20 × 0.20 mm

#### Data collection

Bruker Kappa APEXII CCD diffractometer
12464 measured reflections
3407 independent reflections
2282 reflections with *I* > 2σ(*I*)
*R*_int_ = 0.031

#### Refinement

*R*[*F* ^2^ > 2σ(*F* ^2^)] = 0.045
*wR*(*F* ^2^) = 0.162
*S* = 1.01
3407 reflections
172 parameters
H-atom parameters constrained
Δρ_max_ = 0.20 e Å^−3^
Δρ_min_ = −0.16 e Å^−3^

### 

Data collection: *APEX2* (Bruker, 2004 ▶); cell refinement: *SAINT* (Bruker, 2004 ▶); data reduction: *SAINT* and *XPREP* (Bruker, 2004 ▶); program(s) used to solve structure: *SHELXS97* (Sheldrick, 2008 ▶); program(s) used to refine structure: *SHELXL97* (Sheldrick, 2008 ▶); molecular graphics: *ORTEP-3 for Windows* (Farrugia, 2012 ▶); software used to prepare material for publication: *SHELXL97* and *PLATON* (Spek, 2009 ▶).

## Supplementary Material

Crystal structure: contains datablock(s) I, global. DOI: 10.1107/S1600536813003073/nk2193sup1.cif

Structure factors: contains datablock(s) I. DOI: 10.1107/S1600536813003073/nk2193Isup2.hkl

Supplementary material file. DOI: 10.1107/S1600536813003073/nk2193Isup3.cml

Additional supplementary materials:  crystallographic information; 3D view; checkCIF report

## Figures and Tables

**Table 1:** Hydrogen-bond geometry (Å, °)

*D*—H⋯*A*	*D*—H	H⋯*A*	*D*⋯*A*	*D*—H⋯*A*
O2—H2⋯N1^i^	0.82	2.01	2.8237 (16)	170
C1—H1*A*⋯O2^ii^	0.96	2.51	3.390 (2)	153
C3—H3⋯O3^iii^	0.93	2.51	3.429 (2)	171

Symmetry codes: (i) 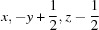; (ii) 
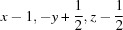
; (iii) 
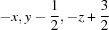
.

**Fig. 1.**
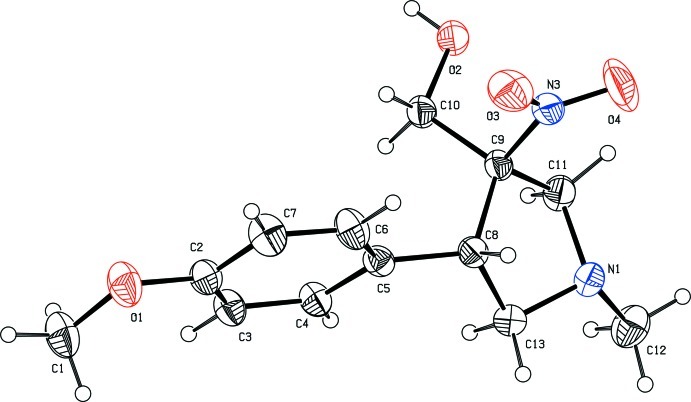
The molecular structure of the title compound, with displacement ellipsoids drawn at the 30% probability level.

**Fig. 2.**
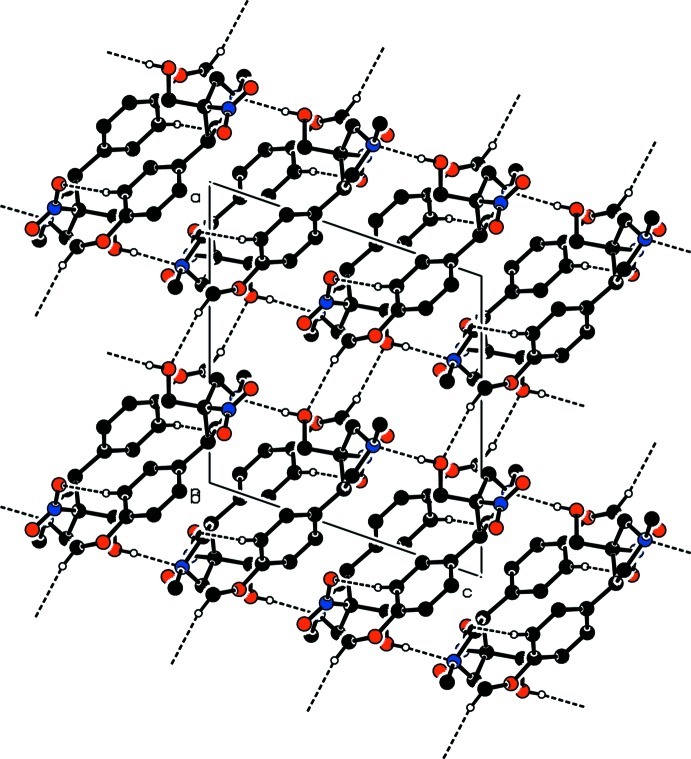
The packing of the molecules in the crystal structure. The dashed lines indicate the hydrogen bonds.

## supplementary crystallographic information

### Comment

The stucture of the title compound, (I), is shown below. Dimensions are available in the archived CIF.

Pyrrolidine ring is present in many biologically active natural compounds and pharmaceuticals (Gu *et al.*, 2004), and find utility in the treatment of diseases such as diabetes (Horri *et al.*,1986), and viral infections (Karpas *et al.*, 1988).

The bond lengths C8—C13 = 1.525 (2) Å; C13—N1=1.460 (2) Å; C11—N1 = 1.462 (2) Å; C11—C9= 1.520 (2) Å; C8—C9= 1.566 (2) Å are longer than the normal values but are comparable with the values of such distances in the reported structure (Jayabharathi *et al.*, 2009). This may be due to the steric forces caused by the bulky group at C8 and C9 of pyrrolidine moiety. C1—O1 [1.416 (3) Å] is longer than C2—O1 [1.367 (2) Å]; this may be due the end atom C1.The dihedral angle between phenyl and pyrrolidine ring is 70.6 (1)°. The sum of angles around N3 [360°] and N1[329.1 (1)°] indicates *sp*^2^and *sp*^3^hybridization, respectively. The five membered ring adopts half chair conformation.The crystal structure is stabilized by intermolecular O—H···N and C—H···O type hydrogen bonds.

### Experimental

Typical Procedure for the synthesis of (E)-3-(4-methoxyphenyl)-2-nitroprop-2-en-1-ol:

To a stirred soln of (E)-1-methoxy-4-(2-nitrovinyl)benzene (10 mmol) in THF (20 mL) at r.t. was added imidazole (1 equiv) followed by anthranilic acid (10 mol%). Aq formaldehyde (38%, 20 mL, excess) was then added and the mixture was stirred at r.t. for the period of 48h. On completion of the reaction (TLC analysis), the mixture was acidified with 5 M HCl (20 mL) and the aqueous layer was extracted with EtOAc (3 × 25 mL). The combined organic layers were washed with brine (50 mL), dried (anhyd Na2SO4), and concentrated in vacuo. The residue was purified by column chromatography (silica gel, EtOAc–hexanes, 0–25%, gradient elution) to afford pure (E)-3-(4-methoxyphenyl)-2-nitroprop-2-en-1-ol in 50% yield as yellow oil.

A mixture of (*E*)-3-(4-methoxyphenyl)-2-nitroprop-2-en-1-ol (2 mmol,0.42 g), *para* formaldehyde (12 mmol,0.36 g) and sacrosine (6 mmol,0.53 g) in acetonitrile(8 ml) was refluxed for 8hrs. After the completion of the reaction as indicated by TLC, the reaction mixture was concentrated and the resulting crude mass was diluted with water (20 ml) and extracted with ethyl acetate (3x10ml) and dried over anhydrous Na_2_SO_4_.The organic layer was concentrated and purified by column chromatography on silica gel (Acme 100–200 mesh), using ethyl acetate:hexane (3:7) to provide the title compound as a colourless solid in 73% (0.39 g) yield.

### Refinement

H atoms were positioned geometrically and treated as riding on their parent atoms, with C—H distance of 0.93 - 0.97 Å, O—H distance of 0.82 Å and *U*_iso_(H) = 1.2Ueq(N,*C*).

### Crystal data

**Table:**

C_13_H_18_N_2_O_4_	*F*(000) = 568
*M**_r_* = 266.29	*D*_x_ = 1.281 Mg m^−^^3^
Monoclinic, *P*2_1_/*c*	Mo *K*α radiation, λ = 0.71073 Å
Hall symbol: -P 2ybc	Cell parameters from 3407 reflections
*a* = 11.6827 (10) Å	θ = 1.5–28.3°
*b* = 11.1912 (11) Å	µ = 0.10 mm^−^^1^
*c* = 11.1789 (11) Å	*T* = 293 K
β = 109.118 (2)°	Block, colourless
*V* = 1381.0 (2) Å^3^	0.22 × 0.20 × 0.20 mm
*Z* = 4	

### Data collection

**Table:**

Bruker Kappa APEXII CCD diffractometer	2282 reflections with *I* > 2σ(*I*)
Radiation source: fine-focus sealed tube	*R*_int_ = 0.031
Graphite monochromator	θ_max_ = 28.3°, θ_min_ = 1.8°
ω and φ scan	*h* = −15→15
12464 measured reflections	*k* = −14→14
3407 independent reflections	*l* = −14→14

### Refinement

**Table:**

Refinement on *F*^2^	Primary atom site location: structure-invariant direct methods
Least-squares matrix: full	Secondary atom site location: difference Fourier map
*R*[*F*^2^ > 2σ(*F*^2^)] = 0.045	Hydrogen site location: inferred from neighbouring sites
*wR*(*F*^2^) = 0.162	H-atom parameters constrained
*S* = 1.01	*w* = 1/[σ^2^(*F*_o_^2^) + (0.1*P*)^2^] where *P* = (*F*_o_^2^ + 2*F*_c_^2^)/3
3407 reflections	(Δ/σ)_max_ < 0.001
172 parameters	Δρ_max_ = 0.20 e Å^−^^3^
0 restraints	Δρ_min_ = −0.16 e Å^−^^3^

### Special details

**Table:**

Geometry. All e.s.d.'s (except the e.s.d. in the dihedral angle between two l.s. planes) are estimated using the full covariance matrix. The cell e.s.d.'s are taken into account individually in the estimation of e.s.d.'s in distances, angles and torsion angles; correlations between e.s.d.'s in cell parameters are only used when they are defined by crystal symmetry. An approximate (isotropic) treatment of cell e.s.d.'s is used for estimating e.s.d.'s involving l.s. planes.
Refinement. Refinement of *F*^2^ against ALL reflections. The weighted *R*-factor *wR* and goodness of fit *S* are based on *F*^2^, conventional *R*-factors *R* are based on *F*, with *F* set to zero for negative *F*^2^. The threshold expression of *F*^2^ > σ(*F*^2^) is used only for calculating *R*-factors(gt) *etc*. and is not relevant to the choice of reflections for refinement. *R*-factors based on *F*^2^ are statistically about twice as large as those based on *F*, and *R*- factors based on ALL data will be even larger.

### Fractional atomic coordinates and isotropic or equivalent isotropic displacement parameters (Å^2^)

**Table:**

	*x*	*y*	*z*	*U*_iso_*/*U*_eq_	
C1	−0.36799 (16)	0.15244 (19)	0.51274 (18)	0.0686 (5)	
H1A	−0.4468	0.1772	0.4592	0.103*	
H1B	−0.3743	0.0770	0.5509	0.103*	
H1C	−0.3151	0.1445	0.4631	0.103*	
C2	−0.20810 (13)	0.21887 (15)	0.69433 (15)	0.0481 (4)	
C3	−0.13157 (13)	0.12749 (15)	0.68781 (14)	0.0487 (4)	
H3	−0.1540	0.0751	0.6195	0.058*	
C4	−0.02079 (13)	0.11333 (14)	0.78317 (14)	0.0451 (4)	
H4	0.0301	0.0513	0.7772	0.054*	
C5	0.01580 (12)	0.18913 (13)	0.88680 (13)	0.0397 (3)	
C6	−0.06274 (14)	0.28269 (16)	0.88911 (16)	0.0544 (4)	
H6	−0.0407	0.3355	0.9571	0.065*	
C7	−0.17096 (15)	0.29922 (17)	0.79468 (17)	0.0589 (5)	
H7	−0.2197	0.3641	0.7976	0.071*	
C8	0.13415 (12)	0.17615 (13)	0.99434 (12)	0.0388 (3)	
H8	0.1198	0.2054	1.0708	0.047*	
C9	0.24446 (12)	0.24842 (13)	0.98146 (12)	0.0380 (3)	
C10	0.22476 (13)	0.29756 (15)	0.84980 (13)	0.0443 (4)	
H10A	0.2056	0.2330	0.7885	0.053*	
H10B	0.1576	0.3535	0.8270	0.053*	
C11	0.35084 (14)	0.16266 (15)	1.02668 (14)	0.0484 (4)	
H11A	0.3672	0.1256	0.9555	0.058*	
H11B	0.4232	0.2039	1.0783	0.058*	
C12	0.38953 (16)	−0.03316 (17)	1.12644 (17)	0.0643 (5)	
H12A	0.4701	−0.0119	1.1783	0.096*	
H12B	0.3916	−0.0659	1.0478	0.096*	
H12C	0.3573	−0.0915	1.1697	0.096*	
C13	0.18708 (14)	0.05085 (15)	1.02428 (15)	0.0481 (4)	
H13A	0.1451	0.0054	1.0711	0.058*	
H13B	0.1826	0.0079	0.9475	0.058*	
N1	0.31240 (11)	0.07359 (12)	1.10121 (11)	0.0433 (3)	
N3	0.26471 (14)	0.35622 (12)	1.07021 (12)	0.0509 (4)	
O1	−0.32042 (10)	0.23895 (12)	0.60855 (13)	0.0663 (4)	
O2	0.33114 (10)	0.35553 (12)	0.84933 (10)	0.0578 (4)	
H2	0.3213	0.3832	0.7788	0.087*	
O3	0.18039 (14)	0.42471 (12)	1.05376 (14)	0.0740 (4)	
O4	0.36032 (15)	0.36987 (16)	1.15196 (15)	0.1019 (6)	

### Atomic displacement parameters (Å^2^)

**Table:**

	*U*^11^	*U*^22^	*U*^33^	*U*^12^	*U*^13^	*U*^23^
C1	0.0467 (9)	0.0815 (14)	0.0648 (12)	0.0034 (9)	0.0006 (8)	−0.0089 (10)
C2	0.0367 (7)	0.0534 (10)	0.0510 (9)	−0.0001 (7)	0.0101 (7)	0.0014 (7)
C3	0.0456 (8)	0.0500 (9)	0.0461 (8)	−0.0014 (7)	0.0091 (7)	−0.0101 (7)
C4	0.0396 (8)	0.0449 (9)	0.0480 (8)	0.0002 (6)	0.0107 (7)	−0.0060 (6)
C5	0.0366 (7)	0.0443 (8)	0.0403 (7)	−0.0048 (6)	0.0155 (6)	−0.0034 (6)
C6	0.0462 (9)	0.0618 (11)	0.0532 (9)	0.0018 (8)	0.0138 (7)	−0.0168 (8)
C7	0.0485 (9)	0.0589 (11)	0.0675 (11)	0.0111 (8)	0.0164 (9)	−0.0118 (8)
C8	0.0392 (7)	0.0449 (8)	0.0341 (7)	−0.0050 (6)	0.0147 (6)	−0.0028 (6)
C9	0.0385 (7)	0.0426 (8)	0.0312 (7)	−0.0060 (6)	0.0092 (6)	−0.0026 (5)
C10	0.0414 (8)	0.0549 (9)	0.0348 (7)	−0.0099 (7)	0.0098 (6)	0.0016 (6)
C11	0.0406 (8)	0.0611 (10)	0.0440 (8)	0.0002 (7)	0.0148 (7)	0.0069 (7)
C12	0.0694 (12)	0.0594 (12)	0.0626 (11)	0.0198 (9)	0.0196 (9)	0.0076 (8)
C13	0.0489 (8)	0.0451 (9)	0.0473 (8)	−0.0063 (7)	0.0116 (7)	0.0033 (6)
N1	0.0432 (7)	0.0483 (8)	0.0377 (6)	0.0036 (5)	0.0124 (5)	0.0043 (5)
N3	0.0627 (9)	0.0478 (8)	0.0384 (7)	−0.0138 (7)	0.0114 (6)	−0.0022 (6)
O1	0.0463 (7)	0.0686 (9)	0.0692 (8)	0.0104 (6)	−0.0013 (6)	−0.0085 (6)
O2	0.0480 (6)	0.0810 (9)	0.0414 (6)	−0.0216 (6)	0.0105 (5)	0.0112 (5)
O3	0.0861 (10)	0.0537 (8)	0.0824 (9)	0.0001 (7)	0.0281 (8)	−0.0176 (7)
O4	0.0916 (11)	0.0971 (12)	0.0788 (10)	−0.0172 (9)	−0.0244 (9)	−0.0309 (9)

### Geometric parameters (Å, º)

**Table:**

C1—O1	1.415 (2)	C9—C10	1.5165 (19)
C1—H1A	0.9600	C9—C11	1.520 (2)
C1—H1B	0.9600	C9—N3	1.5303 (19)
C1—H1C	0.9600	C10—O2	1.4035 (17)
C2—O1	1.3666 (18)	C10—H10A	0.9700
C2—C3	1.376 (2)	C10—H10B	0.9700
C2—C7	1.392 (2)	C11—N1	1.4610 (19)
C3—C4	1.390 (2)	C11—H11A	0.9700
C3—H3	0.9300	C11—H11B	0.9700
C4—C5	1.386 (2)	C12—N1	1.467 (2)
C4—H4	0.9300	C12—H12A	0.9600
C5—C6	1.398 (2)	C12—H12B	0.9600
C5—C8	1.5130 (19)	C12—H12C	0.9600
C6—C7	1.369 (2)	C13—N1	1.4571 (19)
C6—H6	0.9300	C13—H13A	0.9700
C7—H7	0.9300	C13—H13B	0.9700
C8—C13	1.525 (2)	N3—O4	1.1989 (19)
C8—C9	1.5676 (18)	N3—O3	1.214 (2)
C8—H8	0.9800	O2—H2	0.8200
			
O1—C1—H1A	109.5	C11—C9—C8	104.53 (12)
O1—C1—H1B	109.5	N3—C9—C8	107.72 (11)
H1A—C1—H1B	109.5	O2—C10—C9	108.55 (11)
O1—C1—H1C	109.5	O2—C10—H10A	110.0
H1A—C1—H1C	109.5	C9—C10—H10A	110.0
H1B—C1—H1C	109.5	O2—C10—H10B	110.0
O1—C2—C3	125.19 (14)	C9—C10—H10B	110.0
O1—C2—C7	115.70 (14)	H10A—C10—H10B	108.4
C3—C2—C7	119.11 (14)	N1—C11—C9	104.48 (11)
C2—C3—C4	120.13 (14)	N1—C11—H11A	110.9
C2—C3—H3	119.9	C9—C11—H11A	110.9
C4—C3—H3	119.9	N1—C11—H11B	110.9
C5—C4—C3	121.74 (14)	C9—C11—H11B	110.9
C5—C4—H4	119.1	H11A—C11—H11B	108.9
C3—C4—H4	119.1	N1—C12—H12A	109.5
C4—C5—C6	116.70 (13)	N1—C12—H12B	109.5
C4—C5—C8	123.84 (13)	H12A—C12—H12B	109.5
C6—C5—C8	119.46 (13)	N1—C12—H12C	109.5
C7—C6—C5	122.22 (15)	H12A—C12—H12C	109.5
C7—C6—H6	118.9	H12B—C12—H12C	109.5
C5—C6—H6	118.9	N1—C13—C8	103.05 (12)
C6—C7—C2	119.99 (15)	N1—C13—H13A	111.2
C6—C7—H7	120.0	C8—C13—H13A	111.2
C2—C7—H7	120.0	N1—C13—H13B	111.2
C5—C8—C13	117.52 (12)	C8—C13—H13B	111.2
C5—C8—C9	116.25 (11)	H13A—C13—H13B	109.1
C13—C8—C9	102.02 (11)	C13—N1—C11	102.69 (11)
C5—C8—H8	106.8	C13—N1—C12	113.98 (14)
C13—C8—H8	106.8	C11—N1—C12	112.41 (12)
C9—C8—H8	106.8	O4—N3—O3	122.84 (16)
C10—C9—C11	113.55 (12)	O4—N3—C9	120.15 (15)
C10—C9—N3	106.59 (12)	O3—N3—C9	117.01 (13)
C11—C9—N3	110.26 (12)	C2—O1—C1	117.86 (14)
C10—C9—C8	114.09 (11)	C10—O2—H2	109.5
			
O1—C2—C3—C4	−177.74 (15)	C11—C9—C10—O2	−57.84 (17)
C7—C2—C3—C4	2.5 (2)	N3—C9—C10—O2	63.75 (15)
C2—C3—C4—C5	0.3 (2)	C8—C9—C10—O2	−177.49 (12)
C3—C4—C5—C6	−1.7 (2)	C10—C9—C11—N1	−144.62 (12)
C3—C4—C5—C8	178.72 (14)	N3—C9—C11—N1	95.86 (13)
C4—C5—C6—C7	0.4 (2)	C8—C9—C11—N1	−19.66 (14)
C8—C5—C6—C7	179.95 (16)	C5—C8—C13—N1	163.40 (11)
C5—C6—C7—C2	2.4 (3)	C9—C8—C13—N1	35.02 (13)
O1—C2—C7—C6	176.40 (16)	C8—C13—N1—C11	−48.96 (14)
C3—C2—C7—C6	−3.8 (3)	C8—C13—N1—C12	−170.81 (12)
C4—C5—C8—C13	−28.9 (2)	C9—C11—N1—C13	42.62 (14)
C6—C5—C8—C13	151.54 (15)	C9—C11—N1—C12	165.54 (13)
C4—C5—C8—C9	92.35 (17)	C10—C9—N3—O4	−115.56 (17)
C6—C5—C8—C9	−87.21 (17)	C11—C9—N3—O4	8.10 (19)
C5—C8—C9—C10	−13.76 (18)	C8—C9—N3—O4	121.60 (16)
C13—C8—C9—C10	115.42 (13)	C10—C9—N3—O3	64.81 (17)
C5—C8—C9—C11	−138.37 (12)	C11—C9—N3—O3	−171.52 (13)
C13—C8—C9—C11	−9.19 (13)	C8—C9—N3—O3	−58.03 (17)
C5—C8—C9—N3	104.35 (13)	C3—C2—O1—C1	7.2 (3)
C13—C8—C9—N3	−126.47 (12)	C7—C2—O1—C1	−173.08 (17)

### Hydrogen-bond geometry (Å, º)

**Table:**

*D*—H···*A*	*D*—H	H···*A*	*D*···*A*	*D*—H···*A*
O2—H2···N1^i^	0.82	2.01	2.8237 (16)	170
C1—H1*A*···O2^ii^	0.96	2.51	3.390 (2)	153
C3—H3···O3^iii^	0.93	2.51	3.429 (2)	171

Symmetry codes: (i) *x*, −*y*+1/2, *z*−1/2; (ii) *x*−1, −*y*+1/2, *z*−1/2; (iii) −*x*, *y*−1/2, −*z*+3/2.
